# Modulation of *Vibrio cholerae* gene expression through conjugative delivery of engineered regulatory small RNAs

**DOI:** 10.1128/jb.00142-24

**Published:** 2024-09-18

**Authors:** Pilar Menendez-Gil, Diana Veleva, Mollie Virgo, Jige Zhang, Rita Ramalhete, Brian T. Ho

**Affiliations:** 1 Department of Biological Sciences, Institute of Structural and Molecular Biology, Birkbeck College, London, United Kingdom; 2 Division of Biosciences, Institute of Structural and Molecular Biology, University College London, London, United Kingdom; University of Chicago, Chicago, Illinois, USA

**Keywords:** *V. cholerae*, sRNAs, modulation gene expression, T6SS, conjugation

## Abstract

The increase in antibiotic resistance in bacteria has prompted the efforts in developing new alternative strategies for pathogenic bacteria. We explored the feasibility of targeting *Vibrio cholerae* by neutralizing bacterial cellular processes rather than outright killing the pathogen. We investigated the efficacy of delivering engineered regulatory small RNAs (sRNAs) to modulate gene expression through DNA conjugation. As a proof of concept, we engineered several sRNAs targeting the type VI secretion system (T6SS), several of which were able to successfully knockdown the T6SS activity at different degrees. Using the same strategy, we modulated exopolysaccharide production and motility. Lastly, we delivered an sRNA targeting T6SS into *V. cholerae* via conjugation and observed a rapid knockdown of the T6SS activity. Coupling conjugation with engineered sRNAs represents a novel way of modulating gene expression in *V. cholerae* opening the door for the development of novel prophylactic and therapeutic applications.

**IMPORTANCE:**

Given the prevalence of antibiotic resistance, there is an increasing need to develop alternative approaches to managing pathogenic bacteria. In this work, we explore the feasibility of modulating the expression of various cellular systems in *Vibrio cholerae* using engineered regulatory sRNAs delivered into cells via DNA conjugation. These sRNAs are based on regulatory sRNAs found in *V. cholerae* and exploit its native regulatory machinery. By delivering these sRNAs conjugatively along with a real-time marker for DNA transfer, we found that complete knockdown of a targeted cellular system could be achieved within one cell division cycle after sRNA gene delivery. These results indicate that conjugative delivery of engineered regulatory sRNAs is a rapid and robust way of precisely targeting *V. cholerae*.

## INTRODUCTION

The Gram-negative bacterium *Vibrio cholerae* is the causative agent of cholera, a diarrheal disease endemic to several developing countries (1, 2). *V. cholerae* can be found in brackish waters and colonizes human hosts after intake of contaminated water. After colonization of the small intestine, it releases cholera toxin (CT), which is responsible for the disease’s characteristic diarrhea (3, 4). *V. cholerae* utilizes different mechanisms to ensure survival and colonization of the gut, including the expression of several virulence factors [CT, toxin-coregulated pilus (TCP)], antibacterial systems [type VI secretion system (T6SS)], quorum sensing, and biofilm formation (5).

The current treatment for cholera is the use of rehydration therapies in conjunction with antibiotic treatment (4); however, the emergence and spread of antibiotic resistance in bacteria have prompted an increased effort to develop alternative strategies to combat bacterial diseases. Such efforts include vaccine development (6), development of inhibitors of quorum sensing (7) or adhesion (8), and use of novel antibacterials like bacteriophages (9) or extracellular contractile injection systems (10). While these approaches can be effective, by seeking to eliminate the pathogen or block their colonization, they create a strong selective pressure for resistant mutants to emerge. An alternative approach likely to be less prone to resistance would be to alter a pathogen’s genetic composition to eliminate its virulence potential rather than the pathogen itself. Indeed, efforts using lysogenic bacteriophages to deliver virulence-neutralizing factors have been successfully demonstrated for *Escherichia coli* and *Shigella* (11, 12). Unfortunately, the molecular biology and bacteriophage toolbox for *V. cholerae* are not as extensive as for *E. coli*. We therefore wondered if an alternative approach for delivering virulence-neutralizing factors, DNA conjugation, could be used instead. Conjugation systems encoded by broad-host range plasmids have long been used to deliver novel genetic information into *V. cholerae* (13), but there are also *Vibrio*-specific conjugative plasmids that can also be used (14). Additionally, conjugation has been employed as a means of delivering toxic genes for killing pathogens (15, 16).

The virulence-neutralizing factors that have been employed are dCas9 (11) and pathogen-specific transcriptional regulators (12). dCas9 has the disadvantage that it requires the expression of a large dCas9 protein in addition to the guide RNA, while using native transcriptional regulators limits the cellular processes that can be targeted. Instead, we elected to use non-coding regulatory small RNAs (sRNAs) to silence target genes, an approach previously described in *E. coli* (17). sRNAs play a key role in post-transcriptional regulation in bacteria. They act by base pairing to target mRNAs and can regulate mRNA expression both positively and negatively. Most commonly, binding of the sRNA to the target mRNA occurs across the ribosome binding site (RBS) inhibiting ribosome binding and translation, but sRNA binding can also be coupled with degradation by double-stranded ribonucleases (18). In *V. cholerae* and other Gram-negative bacteria, RNA chaperone Hfq will help stabilize the sRNA and help it bind its target (19–21), though not all regulatory sRNAs require Hfq for their function (22).

In this study, we have engineered sRNAs based on the sRNA TarB (22) to modulate *V. cholerae* gene expression. Several sRNAs targeting the T6SS were able to successfully knockdown T6SS activity to different degrees with the most effective one knocking down T6SS activity nearly to the level of a gene knockout mutant. We also observed that modifying the recognition sequence in the mRNA did not significantly affect the efficiency of the sRNA knockdown effect so long as the overall secondary structure of the sRNA was preserved. We also employed this sRNA silencing strategy to target other processes in *V. cholerae*, including modulating exopolysaccharide (EPS) production and motility. Lastly, we successfully delivered an sRNA targeting T6SS into *V. cholerae* via conjugation and observed a rapid knockdown of the T6SS activity. Coupling conjugation with engineered sRNAs represents a novel way of modulating gene expression in *V. cholerae* opening the door for the development of novel prophylactic and therapeutic applications.

## MATERIALS AND METHODS

### Strains, plasmids, and growth conditions

The strains and plasmids used in this study are listed in Tables S1 and S2, respectively. *V. cholerae* and *E. coli* strains were grown in Luria Bertani [LB, 10 g/L tryptone, 5 g/L yeast extract (Formedium), and 5 g/L sodium chloride (Fisher Scientific)] broth at 37°C shaking. When necessary, the following antibiotics were added: streptomycin (50 µg/mL) (Apollo Scientific), chloramphenicol (*V. cholerae* 3 µg/mL, *E. coli* 15 µg/mL) (Sigma-Aldrich), and/or gentamicin (10 µg/mL) (Sigma-Aldrich). *E. coli* MFDpir strains were supplemented with 0.28 mM DAP (diaminopimelic acid) (Sigma-Aldrich). When indicated, cultures were supplemented with 0.2% glucose (Fisher Scientific) or 0.2% arabinose (Sigma-Aldrich).

### sRNA design

The sRNA sequences and all relevant characteristics can be found in File S1 and Table S3, respectively. Engineered sRNAs were constructed using TarB sRNA as a scaffold (22). The recognition binding sequence was changed to base pair to the specific target genes. This sequence was further modified to maintain (as much as possible) the secondary structure of the original TarB sequence. The Vienna RNAfold web server (23) was used to predict sRNA secondary structures and VARNA applet for drawing the RNA structure (24). To confirm the sRNA-mRNA interactions, we used the INTaRNAv2 software (25). sRNAs were then synthesized with PCRs using overlapping primers and then cloned into pBAD33 with specific restriction enzymes.

### Plasmid construction

Plasmids were constructed by amplifying inserts by PCR using specific oligonucleotides and the Q5 high-fidelity enzyme (New England Biolabs). PCRs were purified using NucleoSpin Gel and PCR Clean-up Kit (Macherey-Nagel). Plasmids and PCRs were digested with the appropriate restriction enzymes (New England Biolabs), purified, and ligated using Instant Ligase Sticky-end Ligase Mix (New England Biolabs). Ligations were transformed into NEB10β competent cells following the manufacturer recommendations (New England Biolabs). Correct plasmids were verified by PCR and sanger sequencing. They were then introduced into *E. coli* donor strains by electroporation and then into *V. cholerae* strains through conjugation.

### Competition assays

Prey and predators were grown overnight (ON) at 37°C shaking in LB with the appropriate antibiotics. Next day, 1 mL of each culture was centrifuged at 5,000 *g* for 3 min and pellets were resuspended in 1 mL of fresh LB. One hundred microliters of each sample was subcultured into 10 mL of fresh LB and was grown for 2 h 30 min. The subcultures of strains carrying pBAD33 plasmids were grown with 0.2% arabinose (Sigma-Aldrich). Optical density at 600nm (OD_600_) was measured, cultures were centrifuged at 5,000 *g* for 3 min, and cell density was adjusted to OD_600_ = 10. Prey and predators were mixed to a ratio of 1:1, and 5 µL of the mixture was spotted twice (technical replicates) in LB + 0.2% arabinose plates. Plates were incubated at 37°C for 2 h. Then, bacterial spots were cut and resuspended in 1 mL LB. Bacterial suspensions were serially diluted up to 10^−6^, and 5 µL of each dilution was spotted onto selective plates. Plates were incubated at 37°C ON. Next day, colonies were counted, and photos were taken of the plates. At least three biological replicates, each in technical replicates, were performed.

### RNA extraction and quantitative real-time PCR analysis

Strains were grown in LB ON at 37°C shaking with the appropriate antibiotics. Next day, 20 µL of each ON culture was subcultured into two tubes containing 2 mL fresh LB, one containing 0.2% glucose and the other 0.2% arabinose. After 2 h 30 min at 37°C, cells were harvested with a centrifugation step and the number of cells was adjusted with OD_600_ measuring. RNA extraction and cDNA synthesis were performed with an RNeasy Mini Kit (Qiagen) and QunatiTect Reverse Transcription Kit (Qiagen), respectively, following the manufacturer’s recommendations. Quantitative real-time PCR (qRT-PCR) was performed using SYBR green master mix (Fisher Scientific), 7500 Fast Real-Time PCR System machine and software. Specific primers were designed for each sRNA and target and for the housekeeping gene (*dnaB*). Each experiment was done in biological duplicate or triplicate.

### Motility assays

Strains were grown in LB ON at 37°C shaking with the appropriate antibiotics. Next day, OD_600_ was measured, cultures were centrifuged at 5,000 *g* for 3 min, and cell density was adjusted to OD_600_ = 1. One microliter of each sample was spotted in a tryptone [1% tryptone (Fisher Scientific), 0.5% NaCl (Fisher Scientific)], soft 0.3% agar (Formedium) plate containing 0.2% arabinose and 3 µg/mL chloramphenicol. Plates were incubated at 28°C for 20–22 h. Next day, halo diameters were measured, and photos were taken. As a control, all samples were serially diluted, spotted into Streptomycin plates, and incubated at 37°C ON. Next day, colonies were counted to ensure equally amounts of all samples. Each experiment was conducted in technical duplicate and repeated at least four times.

### Visualization of T6SS activity with fluorescence microscopy

Strains were grown in LB ON at 37°C shaking with the appropriate antibiotics. Next day, 20 µL of each ON culture was subcultured into two tubes containing 2 mL fresh LB, one containing 0.2% glucose and the other 0.2% arabinose. After 2 h 30 min at 37°C, 1 µL of each culture was spotted into a microscope slide containing a 1.5% agarose (Fisher Scientific) pad made from phosphate-buffered saline. Once the culture spot was fully absorbed into the agar, 2-min-long time-lapse recordings were made with image acquisition every 20 s. Two to four biological replicates were performed for each experiment. Foci were counted using “Spot counter” while “Find maxima” was used to count cells, both part of the Fiji software package (26). Quantification was done in at least six different time-lapse videos from at least two biological replicates.

### Visualization of sRNA delivery using fluorescence microscopy

Donor and recipient cells were grown in LB ON at 37°C shaking with the appropriate antibiotics. Next day, 20 µL of each was subcultured into 2 mL of fresh LB and was grown for 2 h 30 min. OD_600_ was measured, cultures were centrifuged at 4,500 *g* for 10 min, and cell density was adjusted to OD_600_ = 1.5. Donor and recipient cells were mixed to a ratio of 10:1, and 2 µL of the mating was spot into a microscope slide containing an M9 [M9 salts (Sigma-Aldrich), 500 M mM MgSO_4_, 40 mM CaCl_2_, and 0.4% casamino acids (Formedium) ] + DAP agarose (1.5%) pad. The slide was incubated at 37°C for 30, 60, or 90 min before visualizing the cells under the microscope, as described in the above section. Since *V. cholerae* is sensitive to a decrease in oxygen availability, the coverslip was not added until the incubation time has passed. A minimum of around 400 conjugation events were analyzed per timepoint and sample.

### Microscopy imaging and analysis

Microscopy was done using a Nikon ECLIPSE Ti2 inverted microscope with a CoolED pE4000 illuminator and a Zyla 4.2 Megapixel Camera. Images were recorded using Nikon Elements software and analyzed using the temporal-color code plugin of the Fiji software package (26).

## RESULTS

### Engineered sRNA TarVipA inactivates *V. cholerae* T6SS

**Fig 1:**
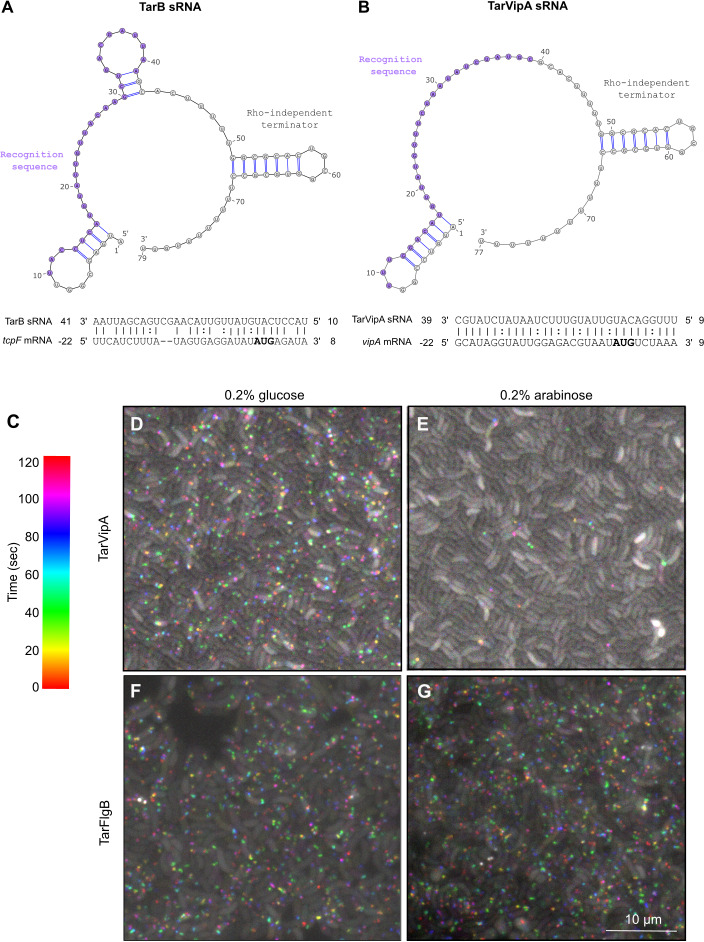
TarVipA sRNA shuts down the T6SS activity of *V. cholerae*. (**A**) TarB secondary structure predicted with Vienna RNAfold web server (23) and TarB and *tcpF* mRNA predicted interaction as stated by Bradley et al. (22). (**B**) TarVipA secondary structure predicted with Vienna RNAfold web server (23) and INTaRNAv2 (25) predicted interaction of TarVipA and *vipA* mRNA. The recognition sequences of TarB to *tcpF* mRNA and TarVipA to *vipA* mRNA are highlighted in purple. *tcpF* and *vipA* start codons are highlighted in bold. The numbering of *tcpF* and *vipA* mRNAs is relative to the start of translation whereas TarB and TarVipA numbering is relative to the start of transcription. (**C**) Spectrum temporal-colored code from ImageJ-Fiji software (26) used to analyze the microscopy in D–G. The temporal code assigns a different color to foci appearing in each time point of the time-lapse. White is assigned to non-dynamic foci/aggregates. (**D - G**) Time-lapse fluorescence microscopy imagining of *V. cholerae* 2740-80 *clpV::clpV-mCherry* carrying pBAD33-TarVipA (**D, E**) or pBAD33-TarFlgB (**F, G**). Cells were either grown in glucose (**D, F**) or in arabinose (**E, G**) to repress or express pBAD33 expression, respectively. Images were taken every 20 s for 2 min. Scale bar is 10 mm for all images shown.

*V. cholerae* employs several non-coding sRNAs as part of its virulence regulation program (20). One of these sRNAs, TarB, is encoded in the *Vibrio* Pathogenicity Island and directly regulates the secreted colonization factor TcpF by binding to the 5′ UTR of the *tcpF* transcript in an Hfq-independent manner (22). The TarB binding target includes the RBS (Fig. 1A) and likely works by blocking TcpF translation. Using the TarB sRNA as a scaffold, we designed novel regulatory sRNAs targeting different biological processes in *V*. *cholerae*. Since the long-term goal of developing deliverable regulatory sRNAs would be to target virulence, our hope was that using virulence-associated TarB as the scaffold would ensure that any cellular machinery needed to utilize these sRNAs would be present and functional in the target bacteria. As an initial proof of concept, we decided to target the T6SS because of the clear and robust reporters for T6SS activity (27, 28). We maintained the Rho-independent terminator (bases 42 to the end) of the original TarB sequence but replaced the target recognition sequence (bases 10 to 41) with a sequence complementary to the equivalent position of a new target gene (Fig. 1B). The positioning of the RBS and the start codon were maintained. Finally, the leader bases at the 5′ end of the transcript (bases 1–9) were changed to generate a small hairpin to match the secondary structure of TarB. For our initial test, we targeted the first gene of the major T6SS gene cluster, *vipA*, and named this sRNA TarVipA accordingly.

TarVipA was cloned into plasmid pBAD33 under the control of an arabinose-inducible promoter and introduced into *V. cholerae* 2740-80 expressing a ClpV-mCherry fusion (28). ClpV-mCherry forms dynamic fluorescent foci as ClpV coalesces onto contracted T6SS sheath structures to facilitate the recycling of secretion apparatus components. In time lapse, these foci manifest as “blinking” spots and can be used as an indicator of T6SS activity (29). Using a temporal color code (Fig. 1C), time-lapse recordings can be projected into a single image enabling the differentiation of dynamic foci (colored spots) from non-dynamic fluorescent aggregates (white spots).

When expression of TarVipA was suppressed (0.2% glucose), colored spots indicating active T6SSs could be observed in nearly every cell (Fig. 1D). However, when the TarVipA was expressed (0.2% arabinose), T6SS activity was almost completely eliminated from the population (Fig. 1E). sRNAs designed to target non-T6SS-related genes resulted in no elimination of T6SS activity, with a representative example, TarFlgB, targeting the *flgB* gene, shown in Fig. 1F and G.

### Significant positional variation in the specific sRNA target site is tolerated

We next wondered how much flexibility there was for designing the specific TarVipA recognition sequence in terms of shifting the recognition sequence relative to the RBS. To address this, we designed six TarVipA variants in which we shifted the recognition sequence 1, 2, or 4 nucleotides upstream (TarVipA_P+1, TarVipA_P+2, and TarVipA_P+4) or downstream (TarVipA_P−1, TarVipA_P−2, and TarVipA_P−4) relative to the original TarVipA target sequence (Fig. S1A). These sRNAs were each cloned into the pBAD33 and introduced into *V. cholerae* 2740-80. Competition assays were performed for each of these *V. cholerae* strains against *E. coli* MG1655 (Fig. S1B and C). Expression of the original TarVipA sRNA and five of the six shifted target sRNAs resulted in almost complete knockdown of T6SS activity. However, there was roughly 1-log less survival of the *E. coli* prey compared with a T6SS genetic deletion consistent with our observation of residual T6SS activity in some cells (Fig. 1E).

One of our shifted target sRNAs, TarVipA_P+2, was unable to knockdown T6SS activity. After closer examination of the predicted secondary structure of this sRNA, we noticed that its recognition sequence was probably sequestered in a larger hairpin structure (Fig. S2). This secondary structure likely prevents TarVipA_P+2 from binding to *vipA* transcript. Overall, these results indicate that sRNA design can tolerate significant shifts in the precise target sequence location, so long as the original secondary structure is preserved.

### The effectiveness of the sRNAs differs depending on the target gene

**Fig 2:**
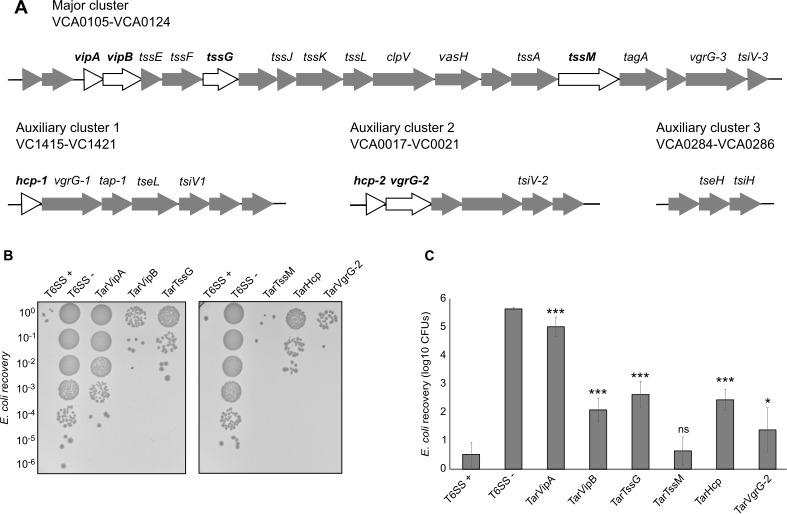
T6SS activity modulation differs depending on the gene being targeted. (**A**) Genetic organization of the T6SS genes (32) with genes targeted by the engineered sRNAs highlighted in white. (**B**) Representative image of the competition assay between *E. coli* MG1655 (prey) and *V. cholerae* 2740-80 *clpV::clpV-mCherry* strains carrying the sRNAs (predators). As a negative control, the predator *V. cholerae* 2740-80 *clpV::clpV-mCherry* (T6SS+) was included. As a positive control, *V. cholerae* 2740-80 *clpV::clpV-mCherry ∆vipA* (T6SS−) was used. (**C**) Quantification of the *E. coli* CFU recovery after competition with *V. cholerae* strains shown in panel B. Data represent the average of at least three independent replicates, each one done in technical duplicate. Error bars represent the standard deviation (SD) of these three replicates. Asterisks represent statistical significance when compared with the T6SS+ no sRNA control (*P value < 0.05 and ***P value < 0.001; ns not significant).

Having successfully knocked down T6SS activity through targeting of *vipA*, we next looked to see if we could similarly knock down T6SS activity by targeting other essential genes both in the main gene cluster and in the auxiliary gene clusters (30, 31) (Fig. 2A). Notably, we wondered if our ability to target *vipA* was dependent on it being the first gene in the operon. To this end, we designed sRNAs targeting *vipB*, *tssG*, *tssM*, *hcp*, and *vgrG-2* (Table S3; File S1), all of which have been previously shown to be essential for T6SS activity (27). Each sRNA was cloned into pBAD33, introduced in *V. cholerae*, and their impact on T6SS activity was measured using competition experiments with *E. coli*. Figure 2B shows a representative image of the *E. coli* MG1655 colony forming units (CFU) recovered (quantified in Fig. 2C). Quantification of *V. cholerae* survival after each competition is included in Fig. S3. Expression of the sRNAs had no impact on *V. cholerae* growth.

Although targeting *vipB*, *tssG*, and *hcp* resulted in a significant knockdown of T6SS activity relative to wild-type T6SS, none was as effective as the TarVipA sRNA. Additionally, targeting *tssM* did not significantly reduce T6SS activity and *vgrG-2* only slightly (Fig. 2B and C). We used qRT-PCR to measure the expression level of the sRNAs (Fig. S4A). While expression of the sRNA targeting *vgrG-2* was poorly expressed, the sRNA targeting *tssM* was expressed to comparable levels as the others. Interestingly, the sRNA targeting *tssG*, which had a significant impact on T6SS activity, was very poorly expressed, suggesting that the expression level may not correlate with the efficacy of the sRNA knockdown. For each sRNA, we also measured the mRNA levels of the target gene via qRT-PCR (Fig. S4B). Although we still observed a decrease in transcript levels, this was not well correlated with T6SS activity. Ultimately, it is not entirely clear why there were differences in the efficacy of the different sRNAs, but considering that the sRNAs were expressed for at least six to seven doubling times, differences in protein turnover rate should not be major a factor. More likely, they are due to variability in the sRNA stability as well as the intrinsic ability of the different sRNAs to access the target site on the mRNA transcript.

### Engineered sRNAs can be used to modulate diverse biological processes in *V. cholerae*

Next, we wondered if other cellular processes could be modulated by similarly engineered sRNAs. EPS is one of the main components of biofilm in *V. cholerae* and plays a major role in transmission and intestinal colonization as well as survival in aquatic environments (33). EPS is synthesized by several proteins encoded in two regions: *vps-I* (coding for *vpsU* and *vpsA-K*) and *vps-II* (coding for *vpsL-Q*) (33, 34). We designed sRNAs targeting three different genes previously shown to be essential for EPS production: *vpsU* (TarVpsU), *vpsA* (TarVpsA), and *vpsL* (TarVpsL) (Table S3; File S1). *vpsU* and *vpsA* are the first two genes of the *vps-I* cluster while *vpsL* is the first gene of the *vps-II* cluster (33, 34).

**Fig 3:**
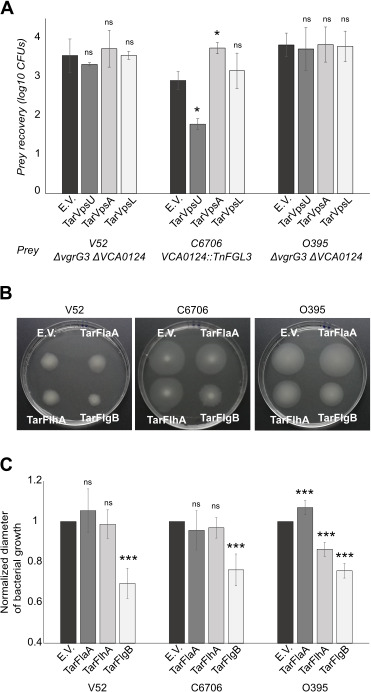
Engineered sRNAs can modulate EPS production and motility in *V. cholerae*. (**A**) Quantification of prey CFUs recovered after the competition assay between *different V. cholerae* strains carrying pBAD33 with sRNAs targeting EPS production (preys) and the *V. cholerae* 2740-80 *clpV::clpV-mCherry* (predator T6SS+). Data represent the average of at least three independent replicates, each one done in technical duplicate. Error bars represent the SD. E.V., empty vector. (**B**) Representative image of the motility assay of different *V. cholerae* strains carrying sRNAs targeting the flagellum apparatus expressed from pBAD33 backbones. (**C**) Quantification of the motility halos (diameter) shown in panel C. Data are represented as a ratio between the halo of a sample in relation to the halo diameter of the empty vector sample (normalized diameter). Data represent the average of at least four independent replicates, each one done in technical duplicate. Error bars represent the SD of these replicates. Asterisks represent statistical significance when compared with the empty vector sample (paired two-tail t-test, *P value < 0.05, **P value < 0.01, and ***P value < 0.001; ns not significant).

EPS formation was previously shown to protect *V. cholerae* from exogenous T6SS attack (35). We used this property to test the efficacy of sRNA knockdown. However, when we transformed each of these sRNAs into *V. cholerae* V52, the prey strain used in that study, we did not observe any effect on T6SS sensitivity (Fig. 3A). Recognizing that *V. cholerae* V52 is an O37 serotype strain with a distinct evolutionary lineage from the *El tor* O1 strains in which TarB has been previously studied, we also introduced each of the sRNAs into a T6SS effector immunity protein knockout mutant of *V. cholerae* C6706. When this strain was mixed with T6SS-active *V. cholerae* 2740-80, two of the sRNAs had a profound impact on their resistance to T6SS-mediated killing (Fig. 3A). Although targeting *vpsL* had no effect, TarVpsU expression significantly increased the sensitivity of C6706 to being killed, indicating successful EPS production knockdown. As a final test, we also introduced the sRNAs into Classical biotype O1 strain, O395, where we also saw no impact on T6SS sensitivity (Fig. 3A). TarVpsU, which had the clearest phenotypic effect on C6706, also had the best expression in that strain (Fig. S4C), though *vpsU* mRNA levels were reduced by tarVpsU in all three strains (Fig. S4D).

Like biofilm formation, motility is another cellular process that plays a role during both *V. cholerae* infection and environmental growth (36). *V. cholerae* has a single sheathed flagellum encoded by multiple genes transcribed in a hierarchal way and located in three large clusters. Motor genes of the flagellum are the exception as they are in three additional loci (37, 38). We designed three sRNAs targeting different flagellar genes: *flaA* (TarFlaA), *flhA* (TarFlhA), and *flgB* (TarFlgB) (Table S3; File S1). FlaA and FlgB are structural components of the filament and basal body, respectively, while FlhA is involved in the export of the flagellar proteins. *flhA* and *flgB* are the first genes of their flagellar operons, while *flaA* is transcribed as a single transcriptional unit (37, 38). We introduced each of the sRNAs into V52, C6706, and O395 and measured their motility on using a tryptone soft agar plate assay (Fig. 3B and C). Strains expressing TarFlgB exhibited less motility relative to the wild type in all three strains, while TarFlhA only affected motility in O395. TarFlaA expression had no effect on V52 or C6706 but slightly increased motility in O395. Similar to the other sRNAs we looked at, there was no clear correlation between sRNA expression levels and their phenotypic impacts (Fig. S4E and F).

Altogether, these results indicate that the efficacy of targeting each specific cellular process will depend on the *V. cholerae* strain. Notably, our engineered sRNAs were most effective in *El tor* O1 strains, where the scaffold TarB sRNA is known to be functional (22, 39).

### Delivery of TarVipA through conjugation results in rapid loss of T6SS activity

Although our engineered sRNAs could successfully knockdown different cellular processes, it was after expression of the sRNA across multiple generations. We next looked to see how quickly after conjugative delivery of an sRNA gene into a given cell could its effects be visible. Since TarVipA had the clearest phenotypic effect among our engineered sRNAs, we focused on this sRNA.

**Fig 4:**
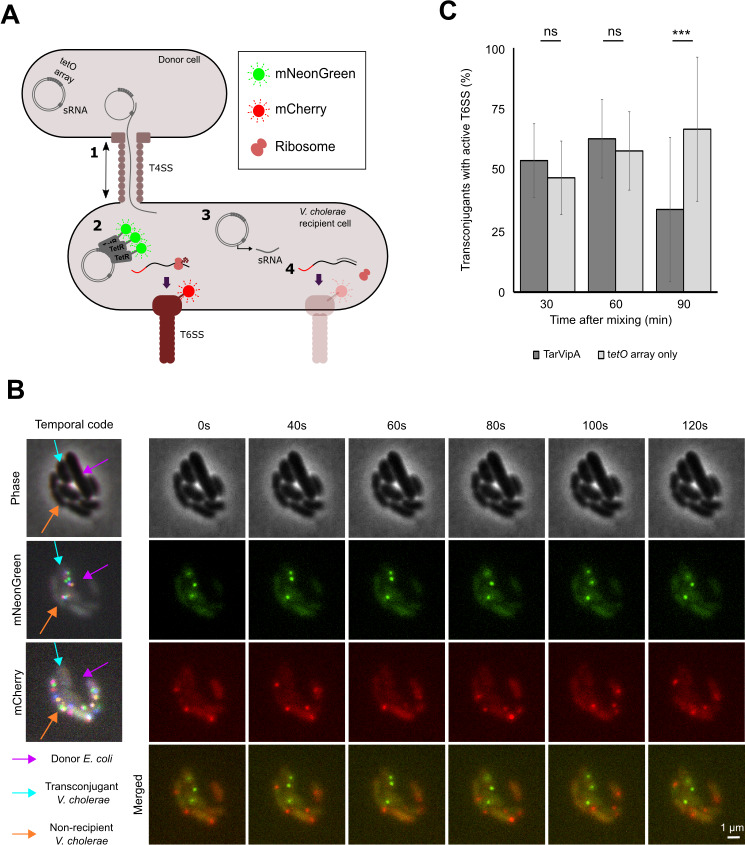
TarVipA rapidly inhibits *V. cholerae* T6SS activity when delivered via conjugation. (**A**) Illustrative image of the assay developed to track conjugation events and T6SS activity with fluorescence microscopy. *E. coli* MFD*pir* donor cells, carrying either pBAD33-TarVipA-*tetO* array or pBAD33-*tetO* array, were mixed with *V. cholerae* 2740-80 *clpV::clpV-mCherry* pBAD33-J23103-tetR-mNeonGreen recipient cells 10:1 and incubated for 30, 60, or 90 min before imaging . First, donors transfer the plasmid carrying the *tetO* array and TarVipA or the tetO array only into a *V. cholerae* strain through the type IV secretion system (T4SS) (1). This *V. cholerae* strain has a ClpV-mCherry fusion to detect T6SS activity. It also expresses a TetR-mNeonGreen fusion, which binds to the *tetO* array, shifting the green fluorescence from being uniformly distributed in the cell to being coalesced into a fluorescent focus (2). Once the plasmid is in the recipient cell, it transcribes the sRNA (3) that will bind to its target mRNA, inhibiting its translation. The sRNA function will be observed by a reduction in the T6SS activity (4). Three biological replicates were conducted for each sRNA delivered. (**B**) Representative image of a 2-min time-lapse microscopy showing the delivery of TarVipA sRNA. An *E. coli* donor can be found surrounded by several *V. cholerae* cells, four of them in direct contact with the *E. coli* cell. Of those four, two were transconjugants (presence of green foci). The red channel shows the presence of active T6SS foci. Merge images are the combination of the red and green channels. Temporal code image analysis of phase, red, and green channels was performed to detect dynamic foci. (**C**) Quantification of cells with an active T6SS after receiving TarVipA or the tetO array only. Statistical significance was determined using student’s t-test, ***P < 0.00001, ns not significant.

We first cloned the sRNA gene into a mobilizable plasmid carrying the oriT region from the IncP plasmid RP4. To track successful conjugative delivery, we also cloned into the plasmid an array of approximately 100 copies of the Tet operator (*tetO)* (40). The cognate repressor TetR, fused to mNeonGreen, was then constitutively expressed in the recipient *V. cholerae* cells. Upon successful delivery of the sRNA plasmid, the TetR-mNeonGreen present in the cell cytosol would bind to the *tetO* array coalescing into a fluorescent focus (Fig. 4A). For the *V. cholerae* strain, we used the 2740-80 *clpV::clpV-mCherry* strain described earlier. When the plasmid carrying the TarVipA and *tetO* array was introduced in *V. cholerae*, although there was still T6SSs in some cells, there was substantially less activity compared with the repressed control (cells grown in glucose) or induction of cells containing the *tetO* array alone (Fig. S5).

To measure the effect of the conjugatively delivered TarVipA on T6SS activity, we mixed a conjugative donor strain of *E. coli* carrying the sRNA plasmid with recipient *V. cholerae* on a microscope slide and then looked for the presence of dynamic clpV-mCherry foci in transconjugant cells containing TetR-mNeonGreen foci (Fig. 4B). After 30 and 60 min of incubation, there was not a significant difference between cells receiving TarVipA and cells receiving the array only control. However, by 90 min, only 34% of the cells that received TarVipA still had an active T6SSs (Fig. 4C). By contrast, roughly 70% of transconjugants receiving the array alone had an active T6SS. This ~40% decrease in T6SS activity after only 90 min shows that conjugative delivery of sRNA can rapidly modulate cellular process in recipient cells.

## DISCUSSION

Overall, this work represents a proof of concept for the use of conjugatively delivered sRNAs to modulate gene expression in *V. cholerae*. We have engineered sRNAs using TarB from *V. cholerae* as a scaffold to modulate T6SS activity, EPS production, and motility. Notably, disruption of these cellular processes required only the expression of the sRNA without the need for delivery and expression of accessory factors, as is needed for other gene silencing strategies previously used in *V. cholerae* (41). The efficiency of gene expression modulation by these sRNAs ranged from completely inhibiting a cellular process (TarVipA) to having nearly no effect (TarVpsL). However, when knockdown of a given process was successful, we observed a large tolerance for variability of the target recognition site. Unless there was an extreme disruption of the original secondary structure, the gene knockdown was equally effective. Additionally, we found no obvious indicators for how effective a given sRNA would be based on the primary sequence or predicted secondary structure. As such, the variability in the effectiveness of our various sRNAs is likely intrinsic to the target genes themselves. This conclusion is further supported by our observation that the same sRNA could have substantially different efficacy in different *V. cholerae* strains. For example, an sRNA targeting EPS production (TarVpsU) was highly effective in the El Tor O1 strain, C6706, but the same sRNA had no effect on the Classical O1 strain, O395. Similarly, an sRNA targeting motility (TarFlhA) was effective in O395 but not in C6706. These strain-specific differences are probably due to differences in the way these systems are regulated in each strain. Therefore, to ensure effective knockdown of a given cellular system, future more practical applications may require concomitant delivery of multiple sRNAs targeting different genes within a given system. That said, such strain-specific effects may actually be advantageous in situations where only a subset of strains in a multi-strain community needs to be targeted.

One advantage of using sRNAs to modulate genes (as opposed to a system like dCas9) is that because it exploits an existing regulatory system, only the delivery and expression of the sRNA itself are required. As such, targeted bacteria do not need to be pre-loaded with accessory regulatory factors, allowing knockdown effects to occur at the rate of protein turnover. In our work, we were able to gain insight into the approximate kinetics of target system modulation by simultaneously tracking conjugative delivery of the sRNA gene and the subsequent shutdown of T6SS activity. We were able to observe sRNA knockdown effects occurring within a single cell division cycle following the conjugation event. Given such rapid response rates, the limiting factor for microbial community modulation will be the rate of sRNA delivery.

The broad-host range conjugative systems employed in this study are an attractive option because they enable cross-species delivery from commensal bacteria into *V. cholerae*. Indeed, contact-dependent antagonistic interactions of *V. cholerae* with resident commensal bacteria mediated by the T6SS have been shown to play a role in *V. cholerae* pathogenesis (42). Given that *V. cholerae* is making direct physical contact with these bacteria, pre-loading them with a conjugative element carrying an anti-T6SS sRNA could be an effective delivery strategy so long as the conjugative element could be stably maintained within the resident population. However, the conjugation rates we observed with our broad-host range conjugative system are probably too low to enable their use for delivery of regulatory sRNAs into larger established communities.

While more efficient delivery may be possible with a Vibrio-specific conjugative plasmid like P factor (14), temperate bacteriophages have already been demonstrated to be an effective delivery tool for *in situ* modification of microbial communities (11, 12). However, the high specificity afforded by phage-based delivery systems necessarily means a narrow range of targetable strains, limiting their use prophylactically. On the other hand, broad-host range conjugative elements can be delivered into a wide range of bacterial species shifting the selectivity and specificity to the sRNA gene and its regulation. As such, incorporating conjugative elements carrying engineered sRNA genes into either probiotic or live vaccine strains should be feasible, but further studies using *in vivo* infection models will be needed to fully assess their efficacy.

## SUPPLEMENTAL MATERIAL

Figure S1 jb.00142-24-s0001.tiff Shifting TarVipA recognition sequence does not impact its efficiency in repressing T6SS activity.

Figure S2 jb.00142-24-s0002.tiff Predicted secondary structure of TarVipA_P+2 sRNA.

Figure S3 jb.00142-24-s0003.tiff Predator control from competition assay.

Figure S4 jb.00142-24-s0004.tiff Expression of sRNAs and their target genes through q-RTPCR analysis.

Figure S5 jb.00142-24-s0005.tiff Time-lapse fluorescence microscopy imagining.

Supplemental File S1 jb.00142-24-s0006.rtf sRNA sequences.

Supplemental tables and figure legends jb.00142-24-s0007.docx Tables S1 to S3; Figure legends for Fig. S1 to S5.

ASM does not own the copyrights to Supplemental Material that may be linked to, or accessed through, an article. The authors have granted ASM a non-exclusive, world-wide license to publish the Supplemental Material files. Please contact the corresponding author directly for reuse.
