# Supplementary figures and images for: Modulation of Vibrio cholerae gene expression through conjugative delivery of engineered regulatory small RNAs
Source: J Bacteriol. 2024 Sep 18;206(10):e00142-24. doi: 10.1128/jb.00142-24 (PMC11500501; doi:10.1128/jb.00142-24)

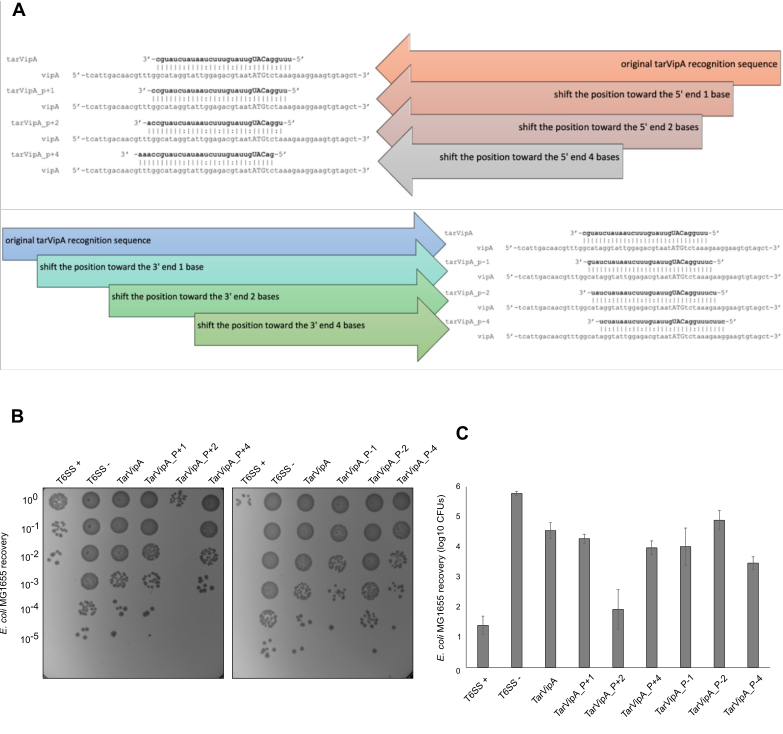

Supplement: Figure S1 — Shifting TarVipA recognition sequence does not impact its efficiency in repressing T6SS activity. [file jb.00142-24-s0001.tiff]

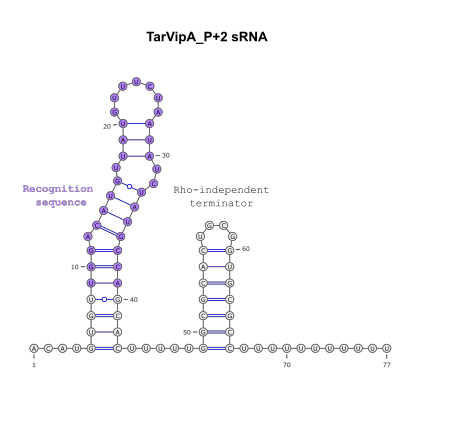

Supplement: Figure S2 — Predicted secondary structure of TarVipA_P+2 sRNA. [file jb.00142-24-s0002.tiff]

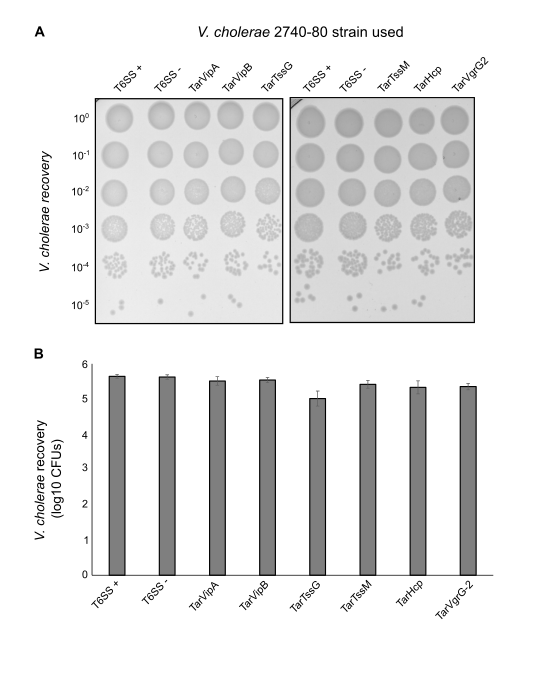

Supplement: Figure S3 — Predator control from competition assay. [file jb.00142-24-s0003.tiff]

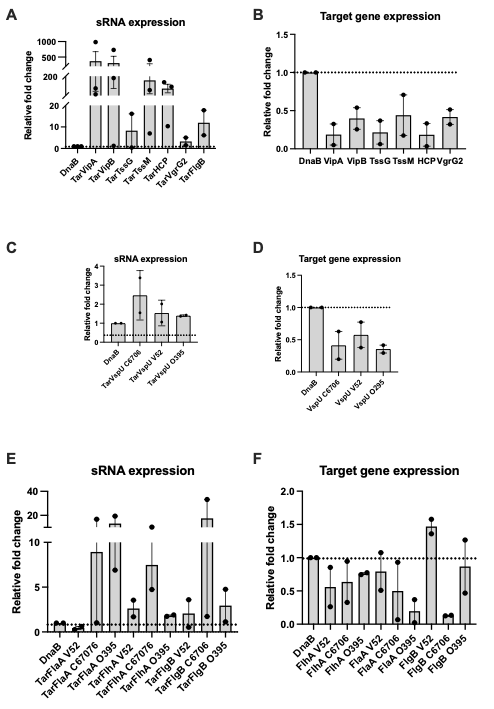

Supplement: Figure S4 — Expression of sRNAs and their target genes through q-RTPCR analysis. [file jb.00142-24-s0004.tiff]

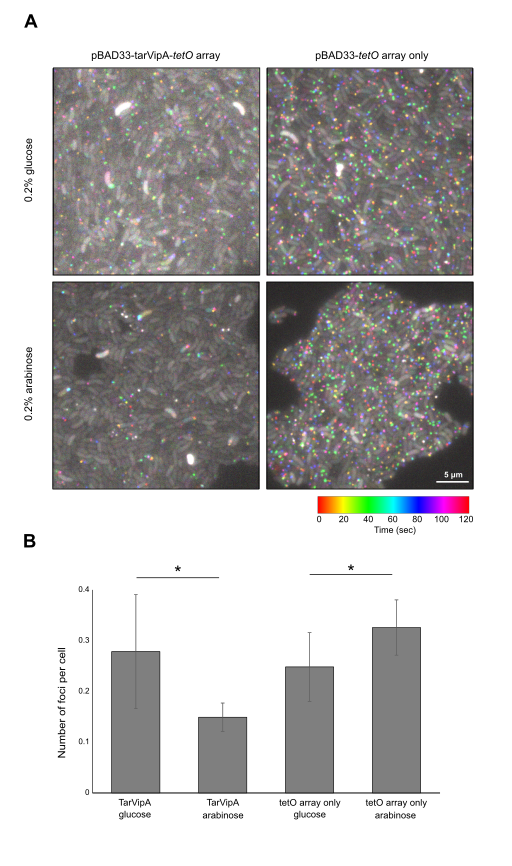

Supplement: Figure S5 — Time-lapse fluorescence microscopy imagining. [file jb.00142-24-s0005.tiff]
