# Supplementary material for: Modulation of Vibrio cholerae gene expression through conjugative delivery of engineered regulatory small RNAs
Source: J Bacteriol. 2024 Sep 18;206(10):e00142-24. doi: 10.1128/jb.00142-24 (PMC11500501; doi:10.1128/jb.00142-24)
Supplement: Supplemental File S1 — sRNA sequences. [file jb.00142-24-s0006.rtf]

>TarVipA
ATGTCCGGTTTGGACATGTTATGTTTCTAATATCTATGCGCACTTTTTGGCGCACTGCGGTGCGCCTTTTTTTTTTT
>TarVipA+1
CATGTCGGTTGGACATGTTATGTTTCTAATATCTATGCCGCACTTTTTGGCGCACTGCGGTGCGCCTTTTTTTTTTT
>TarVipA+2
ACATGTGTTGGACATGTTATGTTTCTAATATCTATGCCAGCACTTTTTGGCGCACTGCGGTGCGCCTTTTTTTTTTT
>TarVipA+4
TAACATCCGACATGTTATGTTTCTAATATCTATGCCAAAGCACTTTTTGGCGCACTGCGGTGCGCCTTTTTTTTTTT
>TarVipA-1
TGTCCACCCTTTGGACATGTTATGTTTCTAATATCTATGGCACTTTTTGGCGCACTGCGGTGCGCCTTTTTTTTTTT
>TarVipA-2
GTCCAACCTCTTTGGACATGTTATGTTTCTAATATCTATGCACTTTTTGGCGCACTGCGGTGCGCCTTTTTTTTTTT
>TarVipA-4
CCAAAGTTCTTCTTTGGACATGTTATGTTTCTAATATCTGCACTTTTTGGCGCACTGCGGTGCGCCTTTTTTTTTTT
>TarVipB
ATGATAGGACATCATTAAGTTCTTAGTGAGTGATTAAGCACTTTTTGGCGCACTGCGGTGCGCCTTTTTTTTTTT
>TarTssG
 ATAGGATGGTGTCCTATGAGTATTTTTCTTGGTTGTTGAGCACTTTTTGGCGCACTGCGGTGCGCCTTTTTTTTTTT
>TarTssM
ATGTAGTGGGTTCTACATGAATTGTGTCTTTGTTTGCTCTGGGAGTTTTTGGCGCACTGCGGTGCGCCTTTTTTTTTTT
>TarHCP
ATGCCATGGGTTGGCATGGCTATTTTCTTTCAGTAAATCCACTTTTTGGCGCACTGCGGTGCGCCTTTTTTTTTTT
>TarVrgG2
ATGGCATGGGTTGCCATCCTGTCTTTCTTTCCTCTACATGCACTTTTTGGCGCACTGCGGTGCGCCTTTTTTTTTTT
>TarVpsT
ATGAAGTGAATCTTCATGCCTTAACTATGCATATGATTTGGAGTTTTTGGCGCACTGCGGTGCGCCTTTTTTTTTTT
>TarVpsA
GTGAAATGGTTTTTCACTTTCCTACATTCTTTTTGAAAAAGCACTTTTTGGCGCACTGCGGTGCGCCTTTTTTTTTTT
>TarVpsL
GTGATATCCTTTATCACTAGTACGCTTCTAACTGATGTAACCACTTTTTGGCGCACTGCGGTGCGCCTTTTTTTTTTT
>TarFlaA
ATAACCTTAGTGGTTATAGTTTGTTCTCTTATTGAGTCGTCTTTTTGGCGCACTGCGGTGCGCCTTTTTTTTTTT
>TarFlhA
ACGAAACGAAATTTCGTAAATTTTTGGTTGTCATTGAAGCACTTTTTGGCGCACTGCGGTGCGCCTTTTTTTTTTT
>TarFlgB
ATGGCTTGGATAGCCATGTTTGCCTTTTCGATAAGAACTCCACTTTTTGGCGCACTGCGGTGCGCCTTTTTTTTTTT
