# Supplementary material for: Modulation of Vibrio cholerae gene expression through conjugative delivery of engineered regulatory small RNAs
Source: J Bacteriol. 2024 Sep 18;206(10):e00142-24. doi: 10.1128/jb.00142-24 (PMC11500501; doi:10.1128/jb.00142-24)
Supplement: Supplemental tables and figure legends — Tables S1 to S3; Figure legends for Fig. S1 to S5. [file jb.00142-24-s0007.docx]

**Table S1 Strains used in this study**

| Strains | Relevant characteristics | Reference/source |
| --- | --- | --- |
| *Escherichia coli* |  |  |
| MG1655 | K12 *E. coli* with *tetO* array introduced in the chromosome. GentR | (1) |
| MFD*pir* | A diaminopimelic acid auxotrophic *E. coli* donor | (2) |
| MFD*pir* pBAD33oriT-TarVipA- *tetO* array | MFD*pir* strain carrying the pBAD33oriT-TarVipA- *tetO* array plasmid | This study |
| MFD*pir* pBAD33oriT- *tetO* array | MFD*pir* carrying the pBAD33oriT-*tetO* array plasmid | This study |
| *Vibrio cholerae* |  |  |
| 2740-80 *clpv::clpv-mCherry* | 2740-80 strain with a *clpV-mCherry* fusion in the *clpV* locus | (1) |
| 2740-80 *clpv::clpv-mCherry ΔvipA* | 2740-80 *clpv::clpv-mCherry* with a deletion of the *vipA* gene | (1) |
| 2740-80 *clpv::clpv-mCherry* pBAD33oriT-TarVipA | 2740-80 *clpv::clpv-mCherry* carrying the pBAD33oriT-TarVipA plasmid | This study |
| 2740-80 *clpv::clpv-mCherry* pBAD33oriT-TarVipA_P+1 | 2740-80 *clpv::clpv-mCherry* carrying the pBAD33oriT-tarVipA_P+1 plasmid | This study |
| 2740-80 *clpv::clpv-mCherry* pBAD33oriT-TarVipA_P+2 | 2740-80 *clpv::clpv-mCherry* carrying the pBAD33oriT-TarVipA_P+2 plasmid | This study |
| 2740-80 *clpv::clpv-mCherry* pBAD33oriT-TarVipA_P+4 | 2740-80 *clpv::clpv-mCherry* carrying the pBAD33oriT-TarVipA_P+4 plasmid | This study |
| 2740-80 *clpv::clpv-mCherry* pBAD33oriT-TarVipA_P-1 | 2740-80 *clpv::clpv-mCherry* carrying the pBAD33oriT-TarVipA_P-1 plasmid | This study |
| 2740-80 *clpv::clpv-mCherry* pBAD33oriT-TarVipA_P-2 | 2740-80 *clpv::clpv-mCherry* carrying the pBAD33oriT-TarVipA_P-2plasmid | This study |
| 2740-80 *clpv::clpv-mCherry* pBAD33oriT-TarVipA_P*-4* | 2740-80 *clpv::clpv-mCherry* carrying the pBAD33oriT-TarVipA_P-4 plasmid | This study |
| 2740-80 *clpv::clpv-mCherry*  *pBAD33*oriT*-TarVipB* | 2740-80 *clpv::clpv-mCherry* carrying the  pBAD33oriT-TarVipB plasmid | This study |
| 2740-80 *clpv::clpv-mCherry*  pBAD33oriT-TarTssG | 2740-80 *clpv::clpv-mCherry* carrying the  pBAD33oriT-TarTssG plasmid | This study |
| 2740-80 *clpv::clpv-mCherry*  pBAD33oriT-TarTssM | 2740-80 *clpv::clpv-mCherry* carrying the  pBAD33oriT-TarTssM plasmid | This study |
| 2740-80 *clpv::clpv-mCherry*  pBAD33oriT-TarHCP | 2740-80 *clpv::clpv-mCherry* carrying the  pBAD33oriT-TarHCP plasmid | This study |
| 2740-80 *clpv::clpv-mCherry*  pBAD33oriT-TarVgrG-2 | 2740-80 *clpv::clpv-mCherry* carrying the  pBAD33oriT-TarVgrG-2 plasmid | This study |
| 2740-80 *clpv::clpv-mCherry* pBAD33oriT-TarVipA- *tetO array* | 2740-80 *clpv::clpv-mCherry* carrying the pBAD33oriT-TarVipA- *tetO* array plasmid | This study |
| 2740-80 *clpv::clpv-mCherry*  pBAD33oriT *tetO* array | 2740-80 *clpv::clpv-mCherry* carrying the pBAD33oriT- *tetO* array plasmid | This study |
| 2740-80 *clpv::clpv-mCherry* pBAD33oriT-J23103-tetR-mNeonGreen | 2740-80 *clpv::clpv-mCherry* carrying the pBAD33oriT-J23103-tetR-mNeonGreen plasmid | This study |
| V52 *Δrhh* | Non pandemic, non O1/O139 strain carrying a deletion of the *rhh* genes | (3) |
| V52 *Δrhh* pBAD33oriT | V52 *Δrhh* carrying pBAD33oriT empty plasmid | This study |
| V52 *Δrhh* pBAD33oriT-TarFlaA | V52 *Δrhh* carrying pBAD33oriT-TarFlaA plasmid | This study |
| V52 *Δrhh* pBAD33oriT-TarFlhA | V52 *Δrhh* carrying pBAD33oriT-TarFlhA plasmid | This study |
| V52 *Δrhh* pBAD33oriT-TarFlgB | V52 *Δrhh* carrying pBAD33oriT-TarFlgB plasmid | This study |
| V52 *Δrhh* *∆VgrG3∆VCA0124* | V52 Δrhh carrying a deletion of *vgrG3* and *VCA0124* genes | (4) |
| V52 *Δrhh* *∆VgrG3∆VCA0124* pBAD33oriT | V52 *Δrhh* *∆VgrG3∆VCA0124* carrying pBAD33oriT empty plasmid | This study |
| V52 *Δrhh* *∆VgrG3∆VCA0124* pBAD33oriT-TarVpsU | V52 *Δrhh* *∆VgrG3∆VCA0124* carrying pBAD33oriT-TarVpsU plasmid | This study |
| V52 *Δrhh* *∆VgrG3∆VCA0124* pBAD33oriT-TarVpsA | V52 *Δrhh* *∆VgrG3∆VCA0124* carrying pBAD33oriT-TarVpsA plasmid | This study |
| V52 *Δrhh* *∆VgrG3∆VCA0124* pBAD33oriT-TarVpsL | V52 *Δrhh* *∆VgrG3∆VCA0124* carrying pBAD33oriT-TarVpsL plasmid | This study |
| C6706 | Pandemic El Tor strain | (5) |
| C6706 pBAD33oriT | C6706 carrying pBAD33oriT empty plasmid | This study |
| C6706 pBAD33oriT-TarFlaA | C6706 carrying pBAD33oriT-TarFlaA plasmid | This study |
| C6706 pBAD33oriT-TarFlhA | C6706 carrying pBAD33oriT-TarFlhA plasmid | This study |
| C6706 pBAD33oriT-TarFlgB | C6706 carrying pBAD33oriT-TarFlgB plasmid | This study |
| C6706 *VCA0124::TnFGL3* | C6706 with the transposon TnFGL3 inserted in the *VCA0124* gene | (5) |
| C6706 *VCA0124::TnFGL3* pBAD33oriT | C6706 *VCA0124::TnFGL3* carrying pBAD33oriT empty plasmid | This study |
| C6706 *VCA0124::TnFGL3* pBAD33oriT-TarVpsU | C6706 *VCA0124::TnFGL3* carrying pBAD33oriT-TarVpsU plasmid | This study |
| C6706 *VCA0124::TnFGL3* pBAD33oriT-TarVpsA | C6706 *VCA0124::TnFGL3* carrying pBAD33oriT-TarVpsA plasmid | This study |
| C6706 *VCA0124::TnFGL3*  pBAD33oriT-TarVpsL | C6706 *VCA0124::TnFGL3* carrying pBAD33oriT-TarVpsL plasmid | This study |
| O395 | Pandemic classical strain | (6) |
| O395 pBAD33oriT | O395 carrying pBAD33oriT empty plasmid | This study |
| O395 pBAD33oriT-TarFlaA | O395 carrying pBAD33oriT-TarFlaA plasmid | This study |
| O395 pBAD33oriT-TarFlhA | O395 carrying pBAD33oriT-TarFlhA plasmid | This study |
| O395 pBAD33oriT-TarFlgB | O395 carrying pBAD33oriT-TarFlgB plasmid | This study |
| O395 *∆VgrG3∆VCA0124* | O395 carrying a deletion of *vgrG3* and *VCA0124* genes | This study |
| O395 *∆VgrG3∆VCA0124* pBAD33oriT | O395 *∆VgrG3∆VCA0124* carrying pBAD33oriT empty plasmid | This study |
| O395 *∆VgrG3∆VCA0124* pBAD33oriT-TarVpsU | O395 *∆VgrG3∆VCA0124* carrying pBAD33oriT-TarVpsU plasmid | This study |
| O395 *∆VgrG3∆VCA0124* pBAD33oriT-TarVpsA | O395 *∆VgrG3∆VCA0124* carrying pBAD33oriT-TarVpsA plasmid | This study |
| O395 *∆VgrG3∆VCA0124* pBAD33oriT-TarVpsL | O395 *∆VgrG3∆VCA0124* carrying pBAD33oriT-TarVpsL plasmid | This study |

**Table S2 Plasmids used in this study**

| Plasmids | Relevant characteristics | Reference/source |
| --- | --- | --- |
| pBAD33oriT | pBAD33 with the origin of transfer from RP4 conjugative plasmid, CmR | This study |
| pBAD33oriT-TarVipA | pBAD33oriT carrying the sRNA TarVipA | This study |
| pBAD33oriT-TarVipA_P+1 | pBAD33oriT carrying the sRNA TarVipA_P+1 | This study |
| pBAD33oriT-TarVipA_P+2 | pBAD33oriT carrying the sRNA TarVipA_P+2 | This study |
| pBAD33oriT-TarVipA_P+4 | pBAD33oriT carrying the sRNA TarVipA_P+4 | This study |
| pBAD33oriT-TarVipA_P-1 | pBAD33oriT carrying the sRNA TarVipA_P-1 | This study |
| pBAD33oriT-TarVipA_P-2 | pBAD33oriT carrying the sRNA TarVipA_P-2 | This study |
| pBAD33oriT-TarVipA_P-4 | pBAD33oriT carrying the sRNA TarVipA_P-4 | This study |
| pBAD33oriT-TarVipB | pBAD33oriT carrying the sRNA TarVipB | This study |
| pBAD33oriT-TarTssG | pBAD33oriT carrying the sRNA TarTssG | This study |
| pBAD33oriT-TarTssM | pBAD33oriT carrying the sRNA TarTssM | This study |
| pBAD33oriT-tarHCP | pBAD33oriT carrying the sRNA TarHCP | This study |
| pBAD33oriT-TarVgrG-2 | pBAD33oriT carrying the sRNA TarVgrG-2 | This study |
| pBAD33oriT-TarVpsU | pBAD33oriT carrying the sRNA TarVpsU | This study |
| pBAD33oriT-TarVpsA | pBAD33oriT carrying the sRNA TarVpsA | This study |
| pBAD33oriT-TarVpsL | pBAD33oriT carrying the sRNA TarVpsL | This study |
| pBAD33oriT-TarFlaA | pBAD33oriT carrying the sRNA TarFlaA | This study |
| pBAD33oriT-TarFlhA | pBAD33oriT carrying the sRNA TarFlhA | This study |
| pBAD33oriT-TarFlgB | pBAD33oriT carrying the sRNA TarFlgB | This study |
| pBAD33oriT-J23103-tetR-mNeonGreen | pBAD33oriT carrying the fusion of the genes *tetR* and *mNeonGreen* under the constitutive promoter J23103 | This study |
| pMOD-2-Lau44T | Carries an array of ~100 copies of t*etO* | This study |
| pBAD33oriT- TarVipA-*tetO array* | pBAD33oriT carrying the sRNA TarVipA and a *tetO* array | This study |
| pBAD33oriT- *tetO* array*T* | pBAD33oriT carrying a *tetO* array | This study |

**Table S3 sRNAs engineered in this study**

| sRNA | Size (bp) | Target mRNA | sRNA binding region (5’-3’)^a^ | mRNA binding region (5’-3’)^b^ |
| --- | --- | --- | --- | --- |
| TarVipA | 77 | vipA (VCA0107) | 9 to 39 | -22 to 9 |
| TarVipA_P+1 | 77 | vipA (VCA0107) | 9 to 39 | -23 to 8 |
| TarVipA_P+2 | 77 | vipA (VCA0107) | 9 to 39 | -24 to 7 |
| TarVipA_P+4 | 77 | vipA (VCA0107) | 9 to 39 | -26 to 5 |
| TarVipA_P-1 | 77 | vipA (VCA0107) | 9 to 39 | -21 to 10 |
| TarVipA_P-2 | 77 | vipA (VCA0107) | 9 to 39 | -20 to 11 |
| TarVipA_P-4 | 77 | vipA (VCA0107) | 9 to 39 | -19 to 13 |
| TarVipB | 75 | vipB (VCA0108) | 8 to 37 | -22 to 8 |
| TarTssG | 77 | *tssG (VCA0111)* | 10 to 39 | -22 to 8 |
| TarTssM | 79 | *tssM (VCA0120)* | 11 to 41 | -23 to 8 |
| TarHCP | 76 | *hcp1 (VC1415) & and hcp-2 (VCA0017)* | 10 to 39 | -22 to 8 |
| TarVgrG-2 | 77 | *vgrG-2 (VCA0018)* | 10 to 39 | -22 to 8 |
| TarVpsU | 77 | *vpsU (VC0916)* | 9 to 39 | -22 to 9 |
| TarVpsA | 78 | *vpsA (VC0917)* | 10 to 41 | -24 to 8 |
| TarVpsL | 78 | *vpsL (VC0934)* | 8 to 40 | -22 to 10 |
| TarFlaA | 75 | *flaA (VC2188)* | 8 to 37 | -20 to 10 |
| TarFlhA | 76 | *flhA (VC2069)* | 9 to 39 | -22 to 9 |
| TarFlgB | 77 | *flgB (VC2200)* | 10 to 39 | -22 to 8 |

^a^ Numbering is relative to the start of transcription.

^b^ Numbering is relative to the start of translation.

**Figure S1. Shifting TarVipA recognition sequence does not impact its efficiency in repressing T6SS activity. (A)** Predicted interaction of TarVipA variants and *vipA* mRNA. **(B)** Representative image of the competition assay between *E. coli* MG1655 and *V. cholerae* 2740-80 *clpV*::*clpV*-*mCherry* carrying the pBAD33-TarVipA variants. As a negative control the predator *V. cholerae* 2740-80 *clpV*::*clpV*-*mCherry* (T6SS +) was included. As a positive control V. cholerae *2740-80* *clpV*::*clpV*-*mCherry* *ΔvipA* (T6SS -) was used. *V. cholerae* recovery is included as a control of equal amounts of predator in all samples. **(C)** Quantification of the *E. coli* CFUs recovered from the competition assay shown in B. Data represent the average of three independent replicates, each one done in technical duplicates. Error bars represent the Standard deviation (SD) of these three replicates.

**Figure S2. Predicted secondary structure of TarVipA_P+2 sRNA.** TarVipA_P+2 secondary structure was predicted with Vienna RNAfold web server (7). The recognition sequence of TarVipA_P+2 to *vipA* mRNA is highlighted in purple.

**Figure S3. Predator control from competition assay shown in Figure 2. A)** Representative image of the V. cholerae (predator) recovery. A competition assay was performed between *E. coli* MG1655 and *V. cholerae* 2740-80 *clpV*::*clpV*-*mCherry* carrying the T6SS sRNAs. As a negative control the predator *V. cholerae* 2740-80 *clpV*::*clpV*-*mCherry* (T6SS +) was included. As a positive control V. cholerae *2740-80* *clpV*::*clpV*-*mCherry* *ΔvipA* (T6SS -) was used. *V. cholerae* recovery is included as a control of equal amounts of predator in all samples. **(C)** Quantification of the *V. cholerae* CFUs recovered from the competition assay shown in B. Data represent the average of three independent replicates, each one done in technical duplicates. Error bars represent the Standard deviation (SD) of these three replicates.

**Figure S4**. **Expression of sRNAs and their target genes through q-RTPCR analysis.** Cells carrying different sRNAs were grown either with glucose (repression) or arabinose (induction). The relative fold change of the sRNA expression and their target genes in these two situations was calculated and normalized to the housekeeping gene *dnaB.* **A*)*** *Fold change in the expression of sRNAs targeting the T6SS. Expressed in V. cholerae* 2740-80 *clpV*::*clpV*-*mCherry*. **B)** Relative fold change in the expression of the target genes of the sRNAs analysed in A. The same samples were used to measure both sRNA and target gene. **C-D)** Fold change in the expression of the TarVpsU sRNA **(C)** and its target gene **(D)** when expressed from three different *V. cholerae* strains, C6706, V52 and O395. **E-F)** Fold change in the expression of the sRNAs targeting the flagellum apparatus **(E)** and their target genes **(F)** when expressed from three different *V. cholerae* strains, C6706, V52 and O395.

**Figure S5. Time-lapse fluorescence microscopy imagining of *V. cholerae 2740-80 clpV::clpV-mCherry* carrying pBAD33oriT-tarVipA-*tetO* array or pBAD33oriT-*tetO* array.** **(A)** Cells were either grown in glucose or in arabinose to repress or express pBAD33 expression, respectively. Images were taken every 20s for 2 min. The imagining was processed with the spectrum temporal-colored code from Image J-Fiji software (8) to assess T6SS activity. Scale bar is 5 μm. **(B)** Quantification of the number of foci for each of the samples stated in (A). The total number of foci was divided by the number of cells to determine the number of foci per cell. Error bars represent the standard deviation of 2 biological independent replicates. Asterisks represent statistical significance when comparing glucose vs arabinose samples (paired two-tail t-test, * p value <0.05,).

REFERENCES

1. M. Basler, J. J. Mekalanos, Type 6 secretion dynamics within and between bacterial cells. *Science* **337**, 815 (2012).

2. L. Ferrieres *et al.*, Silent mischief: bacteriophage Mu insertions contaminate products of Escherichia coli random mutagenesis performed using suicidal transposon delivery plasmids mobilized by broad-host-range RP4 conjugative machinery. *J Bacteriol* **192**, 6418-6427 (2010).

3. S. Pukatzki *et al.*, Identification of a conserved bacterial protein secretion system in Vibrio cholerae using the Dictyostelium host model system. *Proc Natl Acad Sci U S A* **103**, 1528-1533 (2006).

4. T. G. Dong, B. T. Ho, D. R. Yoder-Himes, J. J. Mekalanos, Identification of T6SS-dependent effector and immunity proteins by Tn-seq in Vibrio cholerae. *Proc Natl Acad Sci U S A* **110**, 2623-2628 (2013).

5. D. E. Cameron, J. M. Urbach, J. J. Mekalanos, A defined transposon mutant library and its use in identifying motility genes in Vibrio cholerae. *Proc Natl Acad Sci U S A* **105**, 8736-8741 (2008).

6. M. Dziejman *et al.*, Comparative genomic analysis of Vibrio cholerae: genes that correlate with cholera endemic and pandemic disease. *Proc Natl Acad Sci U S A* **99**, 1556-1561 (2002).

7. A. R. Gruber, R. Lorenz, S. H. Bernhart, R. Neubock, I. L. Hofacker, The Vienna RNA websuite. *Nucleic Acids Res* **36**, W70-74 (2008).

8. J. Schindelin *et al.*, Fiji: an open-source platform for biological-image analysis. *Nat Methods* **9**, 676-682 (2012).
